# Tracking deuterium uptake in hydroponically grown maize roots using correlative helium ion microscopy and Raman micro-spectroscopy

**DOI:** 10.1186/s13007-023-01040-y

**Published:** 2023-07-14

**Authors:** Yalda Davoudpour, Steffen Kümmel, Niculina Musat, Hans Hermann Richnow, Matthias Schmidt

**Affiliations:** Department of Isotope Biogeochemistry, Helmholtz-Centre for Environmental Research - UFZ, Leipzig, Germany

**Keywords:** Confocal Raman micro-spectroscopy, Deuterium, Isotope labeling, Helium ion microscopy, Correlative analysis, Maize, Plant growth, Root

## Abstract

**Background:**

Investigations into the growth and self-organization of plant roots is subject to fundamental and applied research in various areas such as botany, agriculture, and soil science. The growth activity of the plant tissue can be investigated by isotope labeling experiments with heavy water and subsequent detection of the deuterium in non-exchangeable positions incorporated into the plant biomass. Commonly used analytical methods to detect deuterium in plants are based on mass-spectrometry or neutron-scattering and they either suffer from elaborated sample preparation, destruction of the sample during analysis, or low spatial resolution. Confocal Raman micro-spectroscopy (CRM) can be considered a promising method to overcome the aforementioned challenges. The substitution of hydrogen with deuterium results in the measurable shift of the CH-related Raman bands. By employing correlative approaches with a high-resolution technique, such as helium ion microscopy (HIM), additional structural information can be added to CRM isotope maps and spatial resolution can be further increased. For that, it is necessary to develop a comprehensive workflow from sample preparation to data processing.

**Results:**

A workflow to prepare and analyze roots of hydroponically grown and deuterium labeled *Zea mays* by correlative HIM-CRM micro-analysis was developed. The accuracy and linearity of deuterium detection by CRM were tested and confirmed with samples of deuterated glucose. A set of root samples taken from deuterated *Zea mays* in a time-series experiment was used to test the entire workflow. The deuterium content in the roots measured by CRM was close to the values obtained by isotope-ratio mass spectrometry. As expected, root tips being the most actively growing root zone had incorporated the highest amount of deuterium which increased with increasing time of labeling. Furthermore, correlative HIM-CRM analysis allowed for obtaining the spatial distribution pattern of deuterium and lignin in root cross-sections. Here, more active root zones with higher deuterium incorporation showed less lignification.

**Conclusions:**

We demonstrated that CRM in combination with deuterium labeling can be an alternative and reliable tool for the analysis of plant growth. This approach together with the developed workflow has the potential to be extended to complex systems such as plant roots grown in soil.

**Supplementary Information:**

The online version contains supplementary material available at 10.1186/s13007-023-01040-y.

## Background

Plant roots are the major driver of dynamic processes that are shaping the ecology of the rhizosphere during plant growth. Bio-geochemical processes which facilitate the uptake of nutrients by plants in the rhizosphere are essential for plant growth and production [1]. A powerful tool to investigate those processes between plants and their environment is stable isotope labeling in combination with isotope-sensitive (micro-) analytics which provides both, spatial and temporal insights [2, 3]. Deuterium (^2^H), in the following referred to as D, provided in the form of heavy water, (^2^H_2_O), in the following referred to as D_2_O has received attention as a general activity marker. It is non-toxic at lower concentrations in mixtures with H_2_O and can be used as a conservative tracer that is detectable even in low concentrations [4]. The incorporation of D into molecules, bio-synthesized by the plant, can be expected in the presence of D_2_O as every bio-synthesis requires hydrogen from water. Stable isotope labeling using D and subsequent analysis has been used to monitor metabolic activity [5–7] and water flow [8–10] in plants. Both influence the growth rate, and according to Meyer et al. “ha[ve] to be adjusted to the metabolic status” [11]. Current techniques to determine the D label in plants are summarized in Table 1.

**Table 1:** Analytical methods for the detection of deuterium label in plant tissues provided by D_2_O

Method	Type		Purpose		Resolution	Destructive		Reference
	Bulk	Imaging	Water flow	Metabolic activity		Yes	No	
**Mass spectrometry (MS)-based**								
Liquid chromatography-mass spectrometry (LC-MS)	+			+	Depend on extraction technique & volume^1^	+		[6]
Time of flight-secondary ion mass spectrometry (Tof-SIMS), cryo-system		+	+		1.8 μm	+		[8]
Gas chromatography–mass spectrometry (GC-MS)	+			+	Depend on extraction technique & volume^1^	+		[12]
**Nuclear Magnetic Resonance (NMR)-based**								
NMR	+			+	**-**		+	[10]
Magnetic resonance imaging (MRI)		+	+		Voxel: 70*70*2000 µm^3^-43*43*3000 µm^3^ Planar:156*156 µm^2^		+	[13]
**Neutron-based**								
Neutron radiography	+	+	+		Temporal: 1 frame every 20 s		+	[14]
					~ 100 μm/pixel			[15]
Fast neutron tomography	+	+	+		-Time resolution/ tomogram: 1–2 min -Physical spatial resolution:~ 220 μm		+	[16]
**Spectroscopy-based**								
Fourier Transform Infrared Spectrometer (FTIR)-attenuated total reflectance (ATR)	+			+	4 cm^− 1^	+		[10]
Micro-Raman spectroscopy	+	+	+		-Spatial: 2 μm step size -Temporal: 300 ms/point -Spectral: ~3 cm^− 1^		+	[17]

^1^ The spatial resolution depends on the limit of detection and the volume of sample needed for extraction of analytes.

The mass spectrometry (MS) based methods have high sensitivity to the isotope labels in metabolites and can monitor the isotope incorporation [18] yet require extraction of plant tissue. Furthermore, most of these methods lack spatial resolution or are bulk methods that cannot precisely localize the site of a metabolite in plant cells or tissues [18]. In contrast, time-of-flight secondary ion mass spectrometry (ToF-SIMS) is a micro-analytical MS method with a mass resolution high enough to detect isotope labels and a lateral resolution that allows resolving plant cells [8]. However, it is a destructive technique and the plant should be dried, rigid enough to withstand high vacuum conditions, and has to have a reasonably flat surface [8]. On the other hand, nuclear magnetic resonance (NMR) is a non-destructive technique with a higher detection limit than common MS techniques and can provide information about the exact location of the incorporated isotope in the metabolites [18] or structural moieties of the biomass. Though, the spatial resolution of NMR imaging is limited to around 100 × 200 µm^2^ (for NMR flow imaging) and is therefore not sufficient to resolve spatially single plant cells [19]. To resolve a plant tissue at the cellular level, the spatial resolution should be better than 5 μm due to the size of plant cells [20]. Neutron-based analyses are non-destructive and non-invasive techniques that are highly sensitive to hydrogen isotopes, in particular in the form of water [14, 15]. They have been used to acquire tomographic and radiographic data on the water flux in plants [14–16]. However, their spatial resolution is limited to 50–200 μm [21], and to the best of our knowledge they have not been used to track D incorporation into plant biomass, probably because of limited sensitivity. Overall, among methods suitable to detect D incorporation in plants the non-destructive -NMR and neutron-based analyses- cannot be used for sub-cellular analysis on the one hand. On the other hand, the destructive ones, in particular ToF-SIMS, provide high sensitivity and spatial resolution at the same time but are more demanding regarding sample preparation and analysis. Therefore, a non-destructive method that can track the incorporation of D in plants, at a resolution close to ToF-SIMS but does not require drying (necessarily) and ultra-high vacuum compatible samples with surfaces of sub-100 nm roughness (e.g. embedded in resin) could be confocal Raman micro-spectroscopy (CRM). The molecules bio-synthesized by the plant leave fingerprint-like bands in Raman spectra, some of which are typically found in plants and are summarized in Table 2. With regard to D they fall into three categories: (1) not affected because not related to hydrogen, (2) theoretically affected but the hydrogen is in an exchangeable position and will exchange with ambient water such that the bands cannot be used to trace D incorporation, and (3) bands that contain hydrogen in a non-exchangeable position and thus can serve to detect the D label, in particular, these bands are related to CH bonds [6].

**Table 2:** The assignment of Raman bands of plant roots

Band (cm^− 1^)	Chemical bond & type of vibration	Molecule	Reference
376–379	1. Syringyl unit, 2. OH torsion in CH_2_OH	1. Lignin, 2. Cellulose Cellulose	[22] [23]
816	COH (stretching vibration)	Pectin	[24]
856	COC (skeletal mode, α-anomers)	Pectin	[25, 26]
897	β-anomers	Cellulose	[25]
983–987	CC & CO (stretching vibration)	Cellulose	[27, 28]
1002–1005	COOH (stretching vibration)	Pectin	[26]
1092–1098	1. CC & CO (stretching vibration) 2. COC (asymmetric stretching vibration)	Cellulose	1 [27, 28] 2 [22, 26]
1122–1128	C-O-C (symmetric vibration)	Cellulose	[26, 29]
1171–1173	1. CC (stretching vibration), CH (deformation bending) + CO (stretching vibration) 2. CH (in plane deformation vibration) & C-O-C or C-O (stretching vibration) 3. CH (bending vibration)	1. Ferulic/p-coumaric acid 2. Cutin & wax 3. Lignin	1 [27] 2 [22] 3 [22]
1266–1278	1. Aryl-O-CH_3_ & Aryl-O of Aryl-OH, guaiacyl ring with CO group) 2. CH (deformation bending)	1.Lignin: coniferyl aldehyde, ferulic/p-coumaric acid 2. Ferulic/p-coumaric acid	1 [22, 27, 28] 2 [27]
1367–1380	HCC, HCO & HOC (deformation, bending vibration)	Cellulose	[24, 26, 28]
1451–1463	1. O-CH_3_ (deformation vibration), guajacyl-ring (by C = O group), CH_2_ scissoring, C–H bending of OCH_3_and CH_2_ 2. HCH & HOC (deformation vibration)	1. Lignin 2. Cellulose	1 [28, 30] 2 [28]
1599–1606	Phenyl ring (symmetric stretching vibration) Aryl ring (symmetric stretching vibration)	Lignin	[27, 28]
1630	C = C ring (stretching vibration, conjugated)	Ferulic/p-coumaric acid	[27]
1688–1698	C = O (stretching vibration)	Lignin: coniferyl aldehyde, COOH of ferulic/p-coumaric acid	[27]
2895–2898	CH (stretching vibration)	Cellulose	[22, 23]
2935	CH (stretching vibration)	Lignin	[23]

Similar to IR-spectroscopy, CRM is a micro-analytical technique to measure molecular vibration spectra but is in contrast based on Raman-scattering instead of absorption of IR light. The technique is non-destructive, with a sub-micrometer resolution that is sensitive to shifts of characteristic vibrational bands due to the incorporation of heavy isotopes. In particular, CH-bond-related Raman-bands (approx. 2800 to 3100 cm^− 1^) shift tremendously to lower energies (CD approx. 2040 to 2300 cm^− 1^) [31] which makes CRM an appropriate method to measure the incorporation of deuterium into the biomass of plants. D-labeling and CRM have already been used to study the metabolic activity of micro-biological systems [31–36] and to monitor the water flux in plants [17]. To the best of our knowledge, D labeling, as a general metabolic activity marker, together with CRM has not been used to analyze the growth activity of plant roots in a spatially resolved manner.

In order to improve its spatial resolution, CRM can be used correlatively with a high-resolution microscope (multi-modal imaging) to provide laterally resolved chemical information essential for deciphering biochemical processes in roots. Bandara et al. recently demonstrated how multi-modal correlative micro-analysis can be used to study plant roots, soil microbes, and mineral particles in a close-to-native state [37]. A possible choice for the high-resolution modality is the scanning helium ion microscope (HIM). In many aspects it resembles a scanning electron microscope (SEM), but has the advantage of sub-nanometer resolution, large depth-of-field, and, most important, charge compensation such that no metal coating (which interferes with the following CRM analysis) of the sample surface is needed [38]. Using image stitching HIM can be used to obtain large fields of view at a high resolution onto which the chemical maps obtained by CRM can be co-registered. For image-registration of multi-modal microscopy data, Rohde et al. developed the ImageJ/FIJI-based [39] software *Correlia* [40] for which the authors developed a flexible set of rigid as well as deformable co-registration strategies [41].

In this work, we employed D-labeling to analyze the growth of *Zea mays* (maize) roots and explored the sensitivity and resolution of CRM in combination with HIM to localize the label in root sections sampled at different time points. Next, we compared the incorporation of the D label into both, roots and leaves, by elemental analysis-chromium-based high-temperature conversion-isotope ratio mass spectrometry (EA-Cr/HTC-IRMS). Then, the spatial analysis of D labeling by CRM was integrated into a workflow for correlative analysis using HIM to generate micrographs showing the spatial distribution pattern of lignin and D with high resolution. This new combinatory method advances the field of chemical imaging by providing isotope distribution maps of plant tissue and opening up new ways to study plant metabolism and biochemical processes in an unprecedented way with (sub)-µm spatial resolution.

## Results

### Development of a workflow for sample preparation and correlative analysis

The benchmarks for the development of a method for spatially resolved detection of the D isotope label in root sections by CRM using correlative microscopy had been set as:

1. the structural and chemical integrity of the plant root shall be largely maintained during the sample preparation process,
2. the CRM maps shall be correlated with a microscopic technique of lateral resolution down to the nanometer scale,
3. the determined concentrations of D shall pass a test for accuracy and linearity against samples of known concentrations.
4. and the D concentrations determined by CRM in the plant roots shall be in reasonable agreement with those obtained by mass spectrometry.

**Fig. 1:**
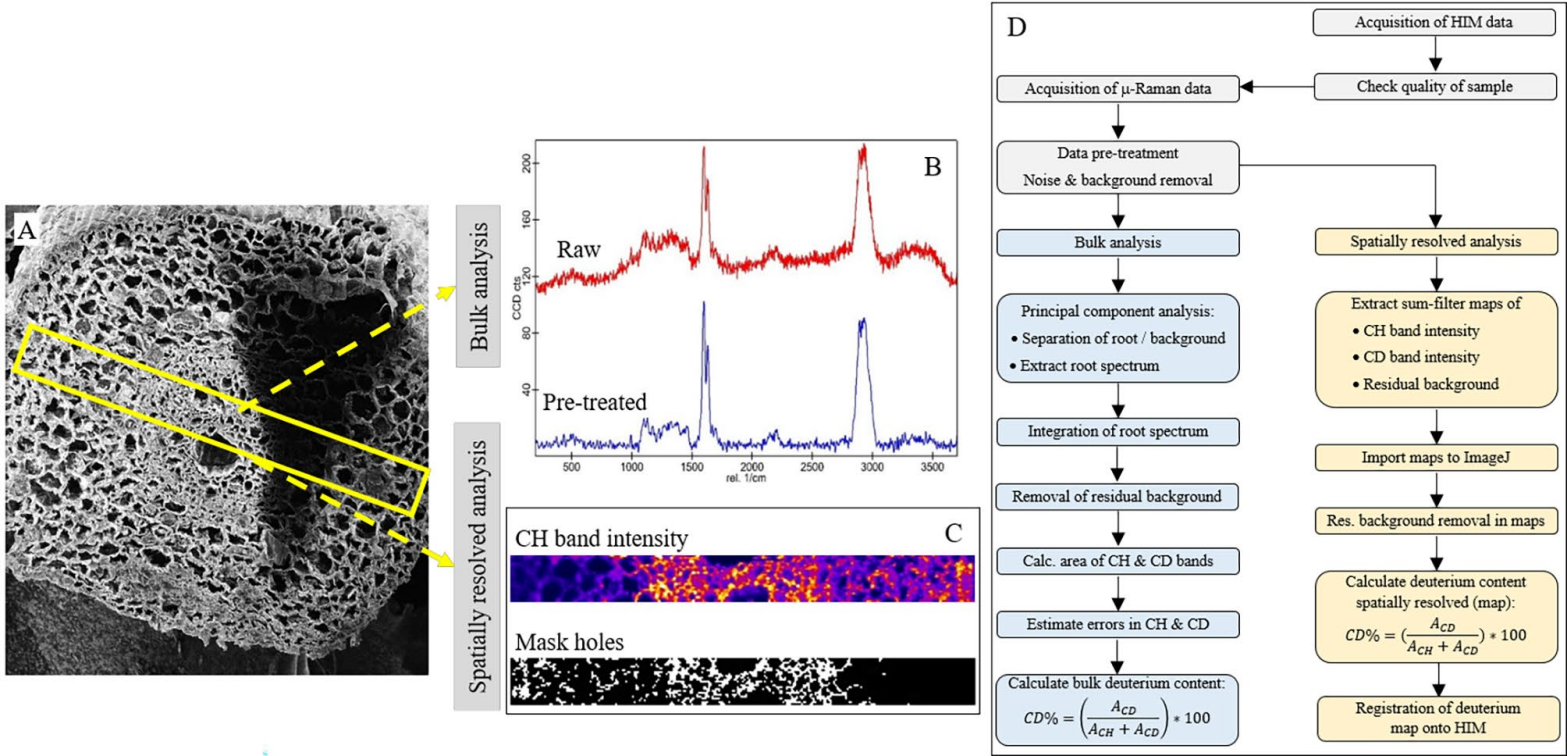
Data acquisition and processing steps. **A** Root cross-section imaged by HIM, yellow rectangle indicates the area analyzed by CRM. **B** Root spectrum obtained from CRM (red: untreated, blue: background, and cosmic ray removed). **C** CRM map of the analyzed area showing CH band (heat map, yellow enriched area) and derived mask to segment map into root (white) and holes/background (black). **D** Workflow for correlative CRM-HIM analysis and data treatment

Considering these benchmarks, the workflow displayed in Fig. 1 has been developed and shall be outlined in the following.

### Preparation of the root sample

The roots were grown hydroponically in Hoagland solution for 16–19 days, then exposed to 40% deuterium oxide, and finally analyzed. The samples grown and analyzed in this work differed in part of the root they were taken from (and thus the root age) and the period they were grown in 40% D_2_O containing Hoagland-solution. In particular, three different root zones, namely the young root as root tip (**Y**), the middle part of the root (**M**), and the old part of the root (**O**) were used. The number of days (*n*) that the sample was exposed to 40% D_2_O containing Hoagland solution (**D**), in other words, the period of D-labeling, is reflected in the sample name **DT*n***. **N** refers to the samples grown with non-deuterated water. The numbers 1, and 2 after the dash stand for replicate 1 and 2. Therefore, samples are labeled as follows, for example, YDT1-2 refers to the young root zone harvested after 1 day of deuterium exposure and 2 for the replicate. Prior to analysis, the root samples were chemically fixed, dehydrated, and gently dried to make them compatible with the high-vacuum conditions in the analysis chamber of the HIM. At the same time this procedure makes them robust enough for subsequent cutting by vibratome as described in the section *Methods*. After a visual inspection by an optical microscope, the vibratome-cut cross-sections were ready for HIM analysis.

### Structural analysis by HIM

HIM imaging of the root cross-sections was done in order to obtain a map that is rich in structural detail onto which the CRM maps could later be registered. Although HIM is known to have a lateral resolution better than 1 nm this workflow does not take immediate advantage of this. Instead, HIM was chosen because of its about five times greater depth-of-field and the possibility of charge compensation by an electron-flood-gun such that, in contrast to SEM, HIM does not require a metal coating of electrically non-conductive-samples (as would be the case for the roots). If the obtained HIM micrograph of a root cross-section was found to be structurally intact, in particular with regard to smoothly cut cell walls and the absence of knife marks, the sample was ready for CRM analysis.

### CRM analysis

#### Data pre-treatment

With regard to the integrity of the plant cells, a strip of 400–600 μm width across the entire cross-section of the root was defined based on the HIM image and scanned by CRM as depicted in Fig. 1. In doing so, for each pixel (size: 2) a Raman spectrum was recorded. As these raw data contain noise as well as background due to root auto-fluorescence and an offset of the CCD camera, they had to undergo a pre-treatment. It comprises the removal of outliers (mostly due to cosmic rays), background, and noise-reduction which was done using WITec Project Five plus software. Cosmic ray removal (CRR) was performed with a threshold-based spectral filter [42] of width 2 (CCD channels) and a dynamic factor of 8. Because of the non-homogeneously distributed auto-fluorescence of the roots a mere subtraction of the dark spectrum of the CCD does not suffice for background removal. Instead, a shape-based (rolling ball) [43] mathematical background subtraction with a spectral shape size of 100 cm^-1^ and noise factor 1 was used. Finally, the noise was removed from the spectra using a 4th -order polynomial with 11 channels Savitzky-Golay filter.

In the next step in the workflow, the data can be evaluated in two different directions. Firstly, it is interesting to determine the global D content from the CRM map, in the following referred to as *bulk analysis*, in order to compare with mass-spectrometry-based bulk methods, here EA-Cr/HTC-IRMS. Secondly, maps of the D concentration in the sample were extracted from the spectral data, in the following referred to as *spatially resolved analysis*.

### Segmentation of CRM data

The concentration of D obtained from CH band shifts in a single Raman spectrum has been widely reported [31, 33, 44, 45]. In order to obtain this number from the CRM maps measured on the root cross-sections, either as a single number for the entire root or for each pixel, it is necessary to segment the CRM map into two classes considered *root* and *others* (e.g. voids which are the empty space inside the cells). In other words, it is needed to de-mask the voids such that only spectra acquired on the cell walls are considered for the calculation of the root spectrum.

#### Bulk analysis

For obtaining a single number for the D content in the root, the CRM map was segmented with the *True Component Analysis* (TCA) module in the WITec Project FIVE plus software. TCA is a base analysis algorithm to detect the main components of the sample by a linear combination of the greatly dissimilar spectra [46]. TCA includes three steps finding components, averaging spectra to obtain a noise-free spectrum, and de-mixing to get as much as possible a pure root spectrum. The obtained Raman spectrum can be considered as an averaged root spectrum and was in the next step extracted to a spreadsheet software for further analysis whilst the void spectrum was discarded.

#### Spatially resolved analysis

In order to obtain the D concentration spatially resolved, the spatial intensity maps of CD, CH, and residual background were extracted by applying sum filters between 2040 and 2300, 2800–3100, and 2450–2650 cm^-1^, respectively, to the pre-treated data. These filters integrate the spectra over the selected wavenumber range. To investigate whether there is a correlation between the lignin content and the deuterium incorporation in the root cross-sections, also the lignin distribution map was extracted with a sum filter in the range of 1599–1606 cm^-1^ [47]. After the residual background had been subtracted from the CD and CH maps, the maps were smoothed for noise reduction with a Gaussian filter (sigma of 0.5). In order to segment the map into the root (cell walls) and void, Otsu thresholding [48] was performed on the sum of the smoothed CH and CD-maps, which contain the structural information of the sample. In doing so, a mask - in essence, a map consisting of pixel values 0 (void) and 1 (root) - was obtained. Later on, this mask-map is multiplied with the CRM maps such that only meaningful pixels (those on the root) remain and are considered for calculations and displaying of the results.

### Calculation of deuterium label content

The amount of D label bound to carbon (CD%) can be obtained from the areas under the CH and CD bands in the CRM spectra and was calculated for both approaches in a similar way. The D content is determined from the shift of the CH-related bands extending from about 2800 to 3100 cm^− 1^ to the range from 2040 to 2300 cm^− 1^ for CD-related bands. Under the assumption that the D label incorporation into the sample is well reflected in these bands, its concentration can be calculated as

1 $$\text{CD}\%=\left( {\frac{{{A_{CD}}}}{{{A_{CH}} + {A_{CD}}}}} \right)*100$$

Where ${A}_{CH}$ and ${A}_{CD}$ are the integrated areas under the respective bands in the Raman spectra. For convenience, and to save computation time, the spectrum is numerically integrated to obtain its primitive integral (in essence the area under the spectrum) using the equation below

2 $${A}_{I}\left(E\right)={\int }_{0}^{E}I\left(\stackrel{-}{E}\right)d\stackrel{-}{E}$$

With the integration margins ${E}_{min}^{CH}<E<{E}_{max}^{CH}$ and ${E}_{min}^{CD}<E<{E}_{max}^{CD}$ for the CH and CD Raman bands, respectively, their areas are calculated by

3 $$\begin{array}{l}{A_{CH}} = {A_I}\left( {E_{max}^{CH}} \right) - {A_I}\left( {E_{min}^{CH}} \right){\rm{and}}\\{A_{CD}} = {A_I}\left( {E_{max}^{CD}} \right) - {A_I}\left( {E_{min}^{CD}} \right)\end{array}$$

After integration of the root spectrum, the primitive integral ${A}_{I}\left(E\right)={\int }_{0}^{E}I\left(\stackrel{-}{E}\right)d\stackrel{-}{E}$ was used to remove residual background by assuming that in the “Raman-silent” region from 2450 to 2650 cm^−1^ the integrated area is zero:

4 $$\begin{array}{c}0 \equiv \int_{2450c{m^{ - 1}}}^{2650c{m^{ - 1}}} {I\left( {\bar E} \right)d\bar E} \\= {A_I}\left( {2650c{m^{ - 1}}} \right) - {A_I}\left( {2450c{m^{ - 1}}} \right),\end{array}$$

Such that a “baseline correction”, as is commonly done, is based on the spectrum itself rather than on the subjective view of the experimentalist.

### Influence of integration ranges for calculation of CD%

The selection of the integration ranges adds a degree of freedom which may have an influence on the calculated values. The well-accepted values proposed in the literature (named here as L-range) are 2040 to 2300 cm^-1^ for the CD band and 2800 to 3100 cm^-1^ for the CH band [31, 33, 44, 45]. The limitation here is that by integrating CRM data using L-ranges the whole band is not covered. Hence, new integration ranges were determined from the acquired data (named A-range) by averaging the CH and CD ranges of all samples. The A-range was 2093–2309 cm^-1^ (CD band) and 2779–3075 cm^-1^ (CH band) for root samples and 2033–2303 cm^-1^ (CD band) and 2665–3045 cm^-1^ (CH band) for deuterated glucose (D-glucose) as test samples. Since the reported values for CD% in this study should be compared with previously reported ones, the calculation of CD% was carried out using both L-range and A-range for D-glucose (see Additional file 1: Fig. S1) and root samples (see Additional file 2: Fig. S2). A very good correlation was found between L-ranges and A-ranges for both D-glucose and roots samples. In the case of roots, among 27 samples, only three samples (ODT2-2, ODT3-1, and YDT4-2) did not follow the line which shows their sensitivity for choosing the range and calculating the deuterium content. Since both ranges showed a positive correlation, the calculated and prepared D-glucose samples were not significantly different, and to be comparable to the available reported CD%, we decided to apply the L-range for the rest of the paper.

### Determination of uncertainty and error propagation

In order to critically evaluate the quality of the CD% values obtained by our method an estimation of their error margins is necessary. On the one hand, the error in the presented data consists of experimental errors resulting, for instance, from slight variations in the environmental condition (e.g. room temperature) that may directly influence the experimental set-up in particular the stability of the excitation laser power. Also, a degradation of the sample under the laser beam, which cannot be fully excluded, would naturally introduce errors. With regard to variations in the laser power, the 532 nm laser has been monitored with a power meter. The variations over 12 h are less than 5% at 5 mW output power. Using the 1582 cm^-1^ Raman line as an indicator for local carbonization of the sample it was found that 5 mW laser power (together with the 20x objective) does not lead to significant deterioration of the root samples.

On the other hand, the obtained accuracy of the determined CD% values depends on the pretreatment of the Raman spectra, namely noise removal and background subtraction, as well as on the choice of the integration limits for the CD and CH bands. The error margins were estimated as follows: firstly, the respective integration limits were increased and their increase of the area under the spectra was regarded as the uncertainty along the energy axis. Secondly, in the same way, the baseline was varied, and its influence was regarded as uncertainty in the intensity. Finally, Gaussian error propagation with these uncertainties was applied to calculate the error margins of the bulk CD% values.

### Calculation of CD%

#### Bulk

**Fig. 2:**
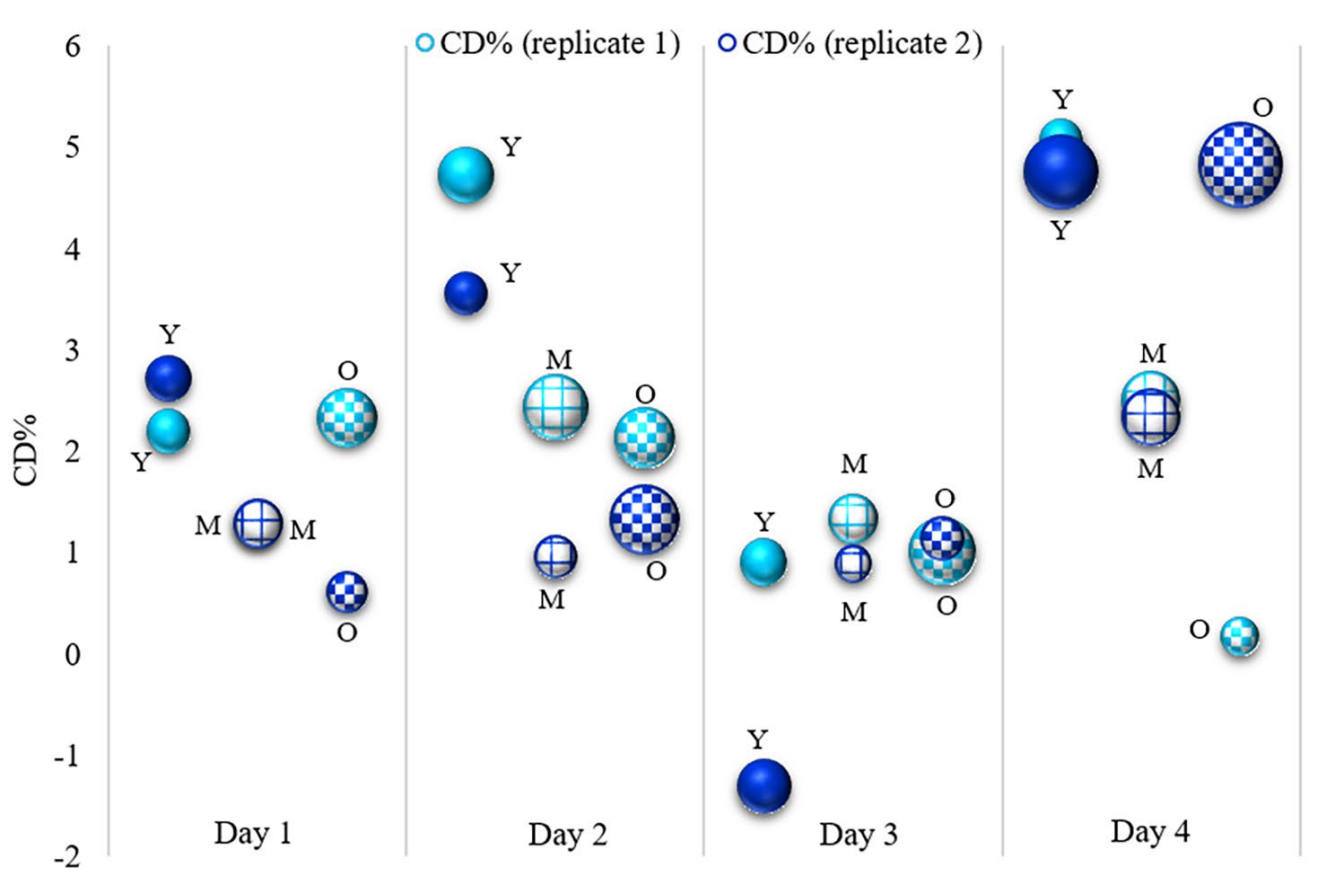
Bulk CD% of Y, M and O root zones calculated from CRM maps. Replicates are shown in different shades of blue. Y, M, and O refer to the young root (root tip), the middle and old part of the root, respectively. Solid fill, grid fill, and checkerboard fill were utilized for Y, M, and O roots, respectively. The bubble size indicates the error in the calculation of CD%. By increasing the time, the CD% increase in Y root zones, except Y roots on day 3. Y roots showed higher CD% (growing activity) compared to M and O root zones. Negative data was obtained from high auto-fluorescence samples and had bad quality data to be analyzed by CRM.

The bulk deuterium content (CD%) calculated from the averaged Raman spectrum of the root (TCA of the segmented CRM map) is depicted in Fig. 2. The reported CD% values were calibrated with the D-glucose trend line (see Additional file 3: Table S1). The bulk CD% values of the roots ranged from close to the natural abundance of D (0.17% in ODT4) to a maximum of 5% in YDT4. Within the error bars of the experiment the CD% values obtained for the different replicates are in good agreement, however, in the case of low label incorporation the accuracy of CD% is low. Generally, on day 1, 2, and 4 of the labeling experiment the CD% in Y roots was higher than in M and O root zones with very similar CD%. Further, the mean CD% (replicates 1 and 2) in the young parts of the roots (Y) increased from day 1 (~ 2.4%) to day 2 (~ 4.1%), then decreased to zero on day 3, and increased again on day 4 (~ 4.9%), see Fig. 2. It is noteworthy that for all replicates and root zones on day 3 of the labeling experiment, CD% was very low which suggests that from day 2 to day 3 the roots did not significantly grow. These results confirmed that the Y roots, being the root tip zones, were actively growing from day 1 to day 4.

#### Spatially resolved

**Fig. 3:**
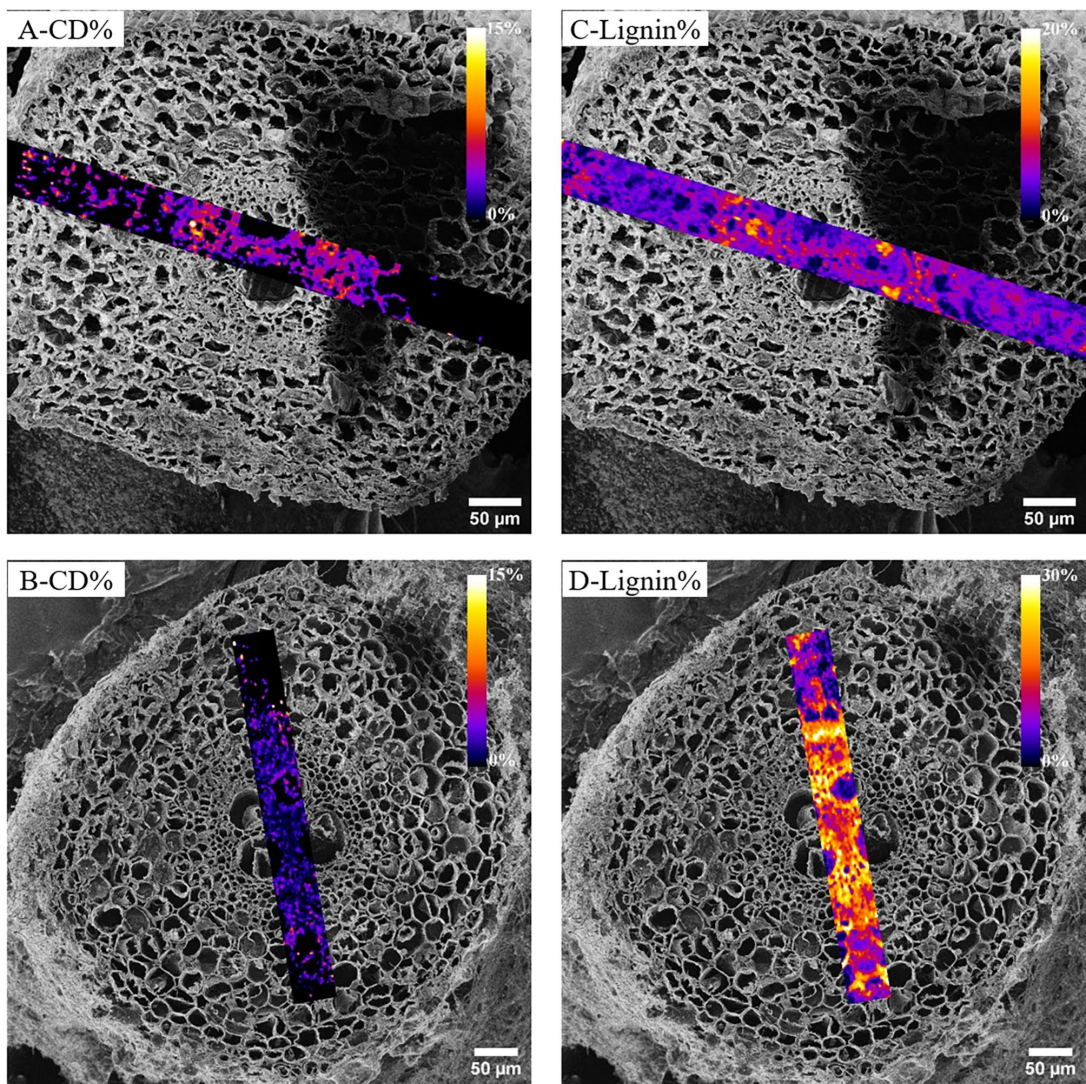
Registered HIM-CRM maps showing the distribution of CD% and lignin% in Y and M roots. The distribution of CD% in YDT2 (**A**) and MDT2 (**B**) as well as the lignin% distribution in YDT2 (**C**) and MDT2 (**D**) are shown in this figure. The heat maps false color-code the distribution of CD% and area of the lignin band normalized on the sum of the CH and CD areas in the Raman spectra. The rectangles show areas mapped by CRM and then registered onto HIM micrographs using the *Correlia* plug-in in ImageJ software. The yellow/white area in A & B panels represents the highest CD%. Both roots accumulated more deuterium in the xylem and phloem than in the cortex. The more mature root MDT2 (**D**) contained much more lignin (yellow color) than the root tip YDT2 (**C**). The shadow effect in panels A & C is most definitely due to the not perfectly flat surface of the root cross-section. We assume that the electron from the electron flood gun did not reach the surface in that area

In order to see how the deuterium label is distributed in the root cross-sections, the lignin% and CD% maps of the YDT2 and MDT2 root cross-sections (as examples) obtained by CRM were registered onto the HIM image of the respective root cross-section (Fig. 3).

As previously mentioned, the lignin content was obtained from CRM maps in the range of 1599 to 1606 cm^− 1^. The deuterium label was mainly distributed in the xylem and phloem of both roots but to a much lesser extent in the cortex (Fig. 3a and b). In both root cross-sections, the central section of the cell wall (xylem and phloem) till the endodermis contained more lignin (Fig. 3c and d); in the MDT2 lignin was also detected in the cortex. Not surprisingly, the more mature root cross-section MDT2 showed much higher lignin content compared to the YDT2 root.

**Fig. 4:**
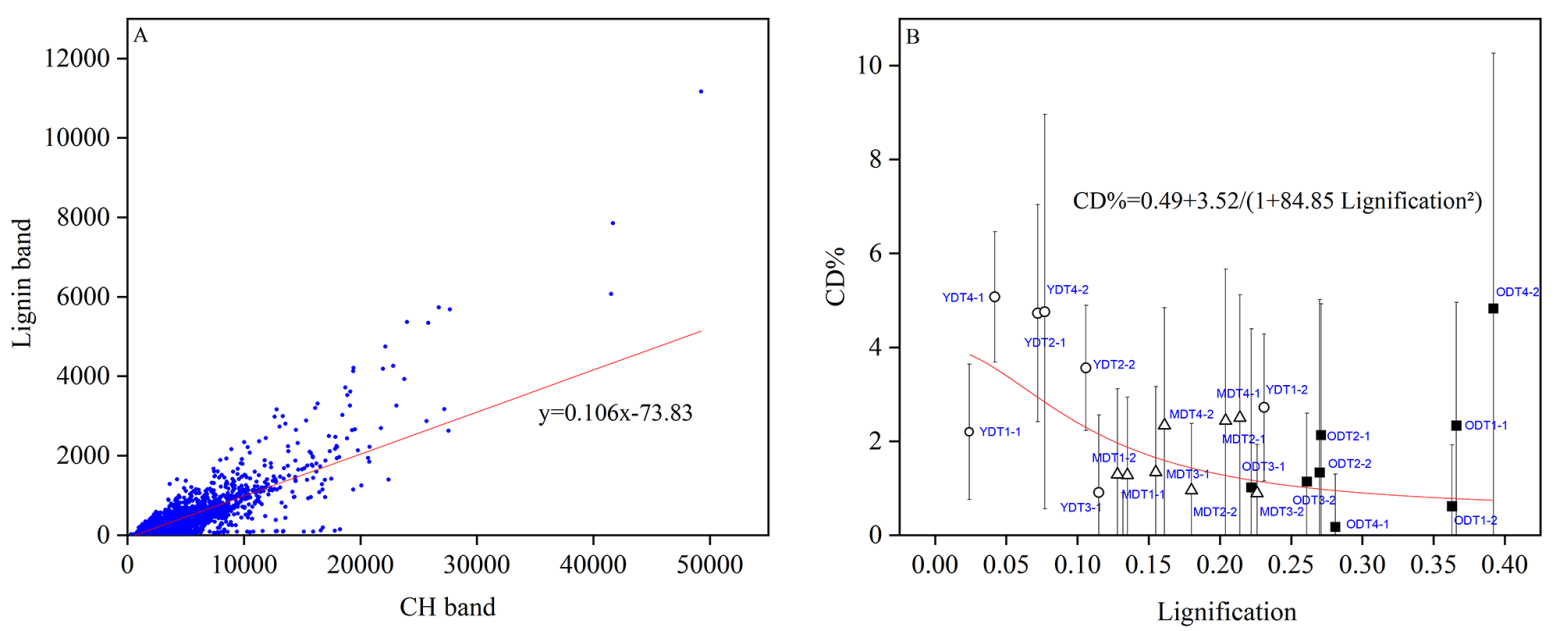
The relation between deuterium incorporation and lignification in the root samples obtained from CRM. **A** Obtaining the lignification from a root cross-section (example: YDT2-2): the area under the lignin band is plotted vs. the area of the CH band, followed by linear regression and reporting the slope as the measure of lignification. **B** CD% vs. lignification plot for Y, M, and O root zones on days 1, 2, 3, and 4. Root zones include Y as the young (root tip), M as the middle, and O as the old part. The circle, triangle, and black-filled square markers were used to indicate Y, M, and O root zones, respectively. Tn is the harvesting time in days after D_2_O incubation. The number after the dash in sample names shows the replicate number. It can be seen that with increasing lignification, CD% decreases

In order to obtain a single number describing the amount of lignin in the CRM-analyzed root cross-sections the quanta ***lignification*** was introduced as follows: The Raman spectra in the CRM scan were analyzed such that the peak area for lignin was plotted (*y*) against the peak area of CH (*x*). Then the data were subjected to linear regression and the slope served as a measure for the lignification of the root (Fig. 4a). Figure 4b illustrates D label incorporation versus lignification for all root zones and at different time points. The data suggest an inverse relation between these two parameters as at low lignification higher CD% was observed and vice versa. Figure 4b also shows that the majority of Y samples have a lignification lower than 0.12 while the majority of M and O samples are higher lignified.

### Image registration

To combine spatially resolved CD% isotopic data with structural information the CD%-CRM map has to be registered onto the corresponding HIM micrograph. For image registration, the ImageJ/FIJI plug-in *Correlia* was used which allows for the registration with landmarks or area similarities, where the first was found to work best for the data sets of this study. In most cases, it will be hard to identify features that occur in both, the HIM micrograph and CD%-CRM map, which can serve as landmarks for the registration. However, if a CRM map with high structural content were available it could be registered onto the HIM micrograph and its coordinates could then serve to register any other CRM map, in particular CD%. With that in mind the CRM map of the CH band - which for all samples resembled very much the shape of the root - was extracted and registered using about ten landmarks. After that, the coordinates of the CD% map were linked to those of the registered CH map such that CD% was registered correctly as well.

### Critical evaluation of the method

#### Testing against a sample of known deuterium content

To evaluate the accuracy of the calculation of CRM data, samples of various percentages of deuterated glucose (D-Glucose) had been prepared from natural D content (not labeled = 0%) to 40% in the laboratory. Since the percentage of deuterium in each sample is known, recalculating it according to the obtained spectrum can be considered as a test for the precision of steps in the workflow. For each sample, Raman spectra were measured at 5 different positions, which were then averaged. The averaged spectrum was analyzed as described above and the deuterium content (CD%) was calculated. The results of calculated bulk CD% and errors in the calculation for various D-glucose samples are reported in Additional file 4: Table S2. A close similarity between the calculated and laboratory-prepared D-glucose was obtained for samples at concentrations of more than 5%. The error in the calculation (ΔCD%) was low for all samples (between 0.017 and 0.135%). After proving the accuracy of steps in the workflow for the data treatment and calculations, CRM data of root samples were evaluated using it.

### Comparison of deuterium content determined by CRM with an EA-Cr/HTC-IRMS

**Fig. 5:**
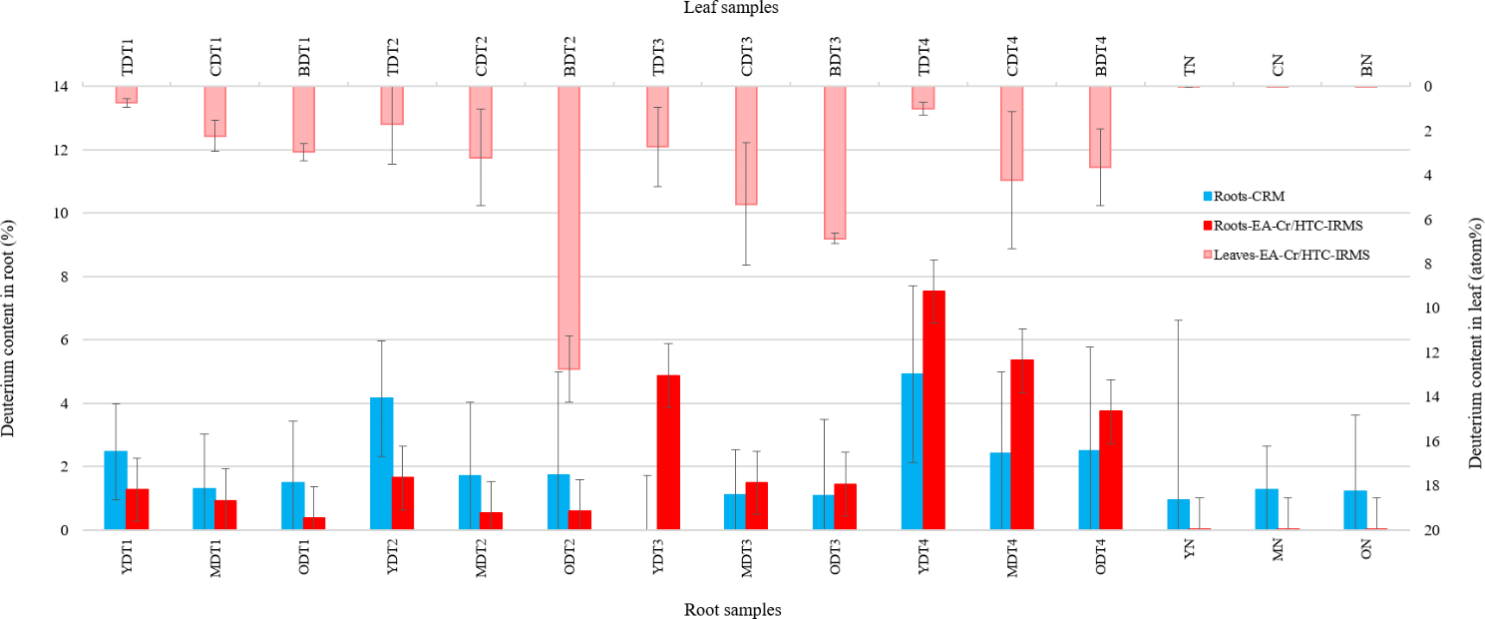
Deuterium content (%) obtained by CRM for roots and by EA-Cr/HTC-IRMS for roots and leaves. Root zones: Y - young (root tip), M - middle, O - old. Leaf zones: T - tip, C - center, B - base. Tn is the time of harvest in days after exposure to D_2_O. N refers to non-labeled samples. Deuterium content in roots was shown by blue and red bars measured with CRM and EA-Cr/HTC-IRMS, respectively. With increasing exposure time, the deuterium content in Y roots increased and was higher than for M and O root zones. The deuterium content measured in the roots by both techniques was comparable. Deuterium content in leaves measured by EA-Cr/HTC-IRMS (light red bars at top) increased from day 1 to day 3 and then decreased on day 4. T zones of leaves represented lower deuterium incorporation than C and B zones. Error bars show error in calculations

In order to evaluate whether CRM data are in agreement with an established method, the deuterium content in the roots was additionally measured by EA-Cr/HTC-IRMS. The deuterium incorporation into roots and leaves was measured by EA-Cr/HTC-IRMS (Fig. 5) and reported as atom% (D/(H + D)).

#### Roots

The deuterium incorporation for root sections continuously increased from day 1 to day 4. The highest deuterium content in root samples was observed in Y root zones, starting from 1.2 atom% on day 1 and gradually increasing over time and reached 7.5 atom% on day 4. For all time points, M root zones showed lower or close deuterium content compared to O root zones, and both, M and O samples contained less D than Y roots (0.36 to 5.3 atom%). With the exception of root sample YDT3, these results are in good agreement with CRM data (Fig. 5). The small difference between deuterium content obtained by CRM and EA-Cr/HTC-IRMS could be due to the dissimilarity of the measurement area by these methods.

#### Leaves

As for the roots, also the leaves were subdivided into zones tip (T), center (C), and base (B) as depicted in Additional file 5: Fig. S3. For almost all analyzed leaf samples the deuterium content in B was higher than in C and decreased further in T (Fig. 5). The determined deuterium concentrations in the leaves ranged from 0.74 to 12.7 atom%. With regard to T and C zones the deuterium content increased from day 1 to day 3 and decreased from day 3 to 4. This trend mostly holds for zone B as well. The only exception is for zone B of day 2 in which the maximum deuterium content was measured.

From day 1 to day 3 the deuterium incorporation in the T and C zones of the leaves is steadily increasing and in the M and O zones of the roots, the incorporation of deuterium is low. With the harvest of day 3, this trend obviously changed: The deuterium content in all zones of the root increased significantly while in all leave sections, the deuterium content dropped on day 4. Obviously, the plant had changed the growth behavior in between these harvests.

## Discussion

### The workflow

A workflow was developed to investigate the growth activity of hydroponically grown maize roots via the incorporation of a D label from water into root biomass over time. The plant was hydroponically grown to keep the system simple and to be in control of the label content. Naturally, the chosen system allows for isolated observation of root growth but lacks the complexity of realistic systems, namely plants grown in soil. An application of the demonstrated workflow to realistic systems may be challenging yet not impossible. The introduction of a UV-bleaching step could help to overcome the auto-fluorescence of soil organic matter (SOM). As no visual difference between the non-deuterated plants and the deuterated ones could be observed it can be concluded that the application of 40% D_2_O did not hamper the growth of the *maize* plant. This observation is in agreement with other reports showing the inhibition of plant roots only growing at concentrations above 50% [10, 49]. No need for extensive sample preparation (e.g. embedding in resin) and comparable analysis time to other spatially resolved isotope-sensitive techniques (e.g., SIMS) are advantages of this workflow. We demonstrated that the laterally resolved measurement of CD% >1% in the plant tissue is achievable with this approach. For this experiment, the pixel size was chosen 2 μm, however, the optical lateral resolution of the microscope was better than 1.5 μm. The workflow was successfully tested for linearity against D-glucose of known concentrations. The obtained deuterium content measured by CRM (CD%) reflects the same trends as EA-Cr/HTC-IRMS (Fig. 5) which shows the second successful test of the workflow. A limitation of the suggested workflow is the necessity to harvest the plant tissue. This is no requirement for some other techniques which can measure the incorporation of deuterium into plant tissue over time without having to harvest it. However, these techniques suffer from low lateral resolution.

### The incorporation of deuterium label into the plant tissue

Deuterium uptake and its incorporation into the plant tissue measured by both, CRM and EA-Cr/HTC-IRMS, indicates the activity and growth of the analyzed part of the plant. It should be mentioned that complete drying is needed before EA-Cr/HTC-IRMS to remove water for precise determination of D incorporation into the biomass. Although for most of the - accurately prepared and dried - root samples EA-Cr/HTC-IRMS and CRM results are in good agreement (Fig. 5), it shall be pointed out that the deuterium content in the roots obtained by these methods can in principle *not* exactly agree. The reason is that the non-homogeneously distributed D in the root cross-sections is differently averaged in CRM and EA-Cr/HTC-IRMS techniques. Whilst in EA-Cr/HTC-IRMS the D in the *entire* root cross-section is averaged, in CRM only a *strip* is analyzed such that the edges (cortex) are under-represented (geometry effect). The difference between deuterium content obtained by CRM and EA-Cr/HTC-IRMS for YDT3 on day 3 can be attributed to the strong auto-fluorescence of the sample which renders the observation of CD bands by CRM impossible but does not affect EA-Cr/HTC-IRMS.

The D content measured in the non-deuterated roots (YN, MN, and ON) by EA-Cr/HTC-IRMS (Fig. 5) was very close to the natural abundance (0.015% [10],). However, CD% measured by CRM in these roots was higher than the natural abundance of D, yet exhibiting large error bars. The reason is mainly the inaccuracy of the CRM data analysis for CD% <1% which results mostly from the imperfect background subtraction from the Raman spectra. Technically, the background subtraction could be improved on the individual spectra, however, the adjustment is small (about 1–2 counts on the CCD) and is difficult to automate, which is a necessity for the treatment of about > 6000 spectra acquired per root cross-section. In the future, potentially machine learning approaches may solve this problem.

As expected, deuterium incorporation, and thus growth of the roots, was observed mostly in the Y samples. Also, older and more mature root zones (M and O) with higher lignin content (lignification > 0.1) are growing, yet less than Y root zones. Obviously, the deuterium content in the roots measured by both techniques, EA-Cr/HTC-IRMS and CRM, was significantly lower than 40%, the D_2_O content in the growth solution. Because of the short period of labeling time (4 days) not only structural units from biosynthesis during this period but also non-labeled structural units from earlier growth phases were used to form new roots and leaves. This observation is in line with previous studies with even longer periods of deuterium labeling: The reported deuterium incorporation into plant biomass grown hydroponically in 50% D_2_O and measured by NMR was 32atom% (for 36 days) and 41atom% (for 172 days) in the stem and leaf of switchgrass [10] and 36.9atom% in annual ryegrass after 61 days [49].

The growth of leaves and roots are parallel processes competing for produced assimilates as well as water and minerals [50]. Therefore, also maize leaves were analyzed for D incorporation by EA-Cr/HTC-IRMS with the result that the deuterium concentration was less than 8atom% with the exception of BDT2. Increasing the incorporated deuterium into the leaves from day 1 to day 3 and then decreasing on day 4 can be explained as follows. For practical reasons, four *maize* plants were grown together in a single pot and one plant after another was harvested from day 1 to day 4. On days 1 and 2 with 4 and 3 plants in the pot, respectively, the plants had grown to a size that they felt the presence of each other such that there was not enough space in the pot for the roots to grow significantly. Therefore, the plants were rather growing leaves - potentially driven by an additional competition for light - which then accumulated more deuterium than the roots. On day 3, only two plants remained in the pot which had developed big roots which in turn accumulated more deuterium than in the days before. On day 4, only one plant was left in the pot. This one grew less in leaves compared to day 3 but had rather a strong root growth possibly due to the availability of even more space after harvesting the plant at day 3.

Overall it can be said, that even during a short period of deuterium labeling up to ~ 8% (CD%) could be measured in the plant tissue rendering deuterium an excellent marker for plant growth that can be detected by CRM.

### Relation between CD% and lignin content in roots

Roots of different ages can show significantly different chemical compositions [51] and with increasing plant age the plant tissue tends to contain more lignin [52]. In this light, the much higher lignin content in the xylem and phloem of middle-aged root section MDT2 compared to the young root-section YDT2 (as shown in Fig. 3) can be explained. On day 2, on which both samples were taken, the plants showed both, leaf and root growth, as shown in Fig. 5. With increasing the size of the plant, the pressure in the stem and tension pressure (enable sap rising) increases [53] and lignin is produced to withstand the negative pressure created during transpiration and water transport [54, 55]. This explains that in MDT2, being an almost mature root zone, lignin is a well-pronounced chemical component in its Raman spectra.

Additionally, Fig. 3 shows a potential relation between the incorporation of deuterium and the lignin band in the Raman-spectrum. Using the before introduced lignification of the root, indeed a weak relation for the entire ensemble of root samples was found as displayed in Fig. 4b. Cutting the root and naming sections according to their age (Y, M, O) is to some extent arbitrary because root length does not necessarily correlate with root age, therefore, the lignification of the root was used as an indicator for its age. Not surprisingly, Y roots were found to be less lignified than M and O roots but showed higher CD% values than those, see Fig. 4b.

In conclusion, we showed here that the proposed workflow can be used to measure and explain major findings of deuterium incorporation into biomass and related growth activity in plant tissue. However, some of the abnormal results obtained in the experiments presented here could most likely be avoided if the labeling experiments were carried out in a separate pot for each plant (making the experiment costlier because of the required amount of heavy water) and for longer labeling times.

## Conclusions

This paper describes a new workflow to determine the incorporation of the growth marker deuterium into roots of hydroponically grown *maize* harvested at different times and root zones. Testing the accuracy of the workflow using D-glucose proved the reliability of the approach. By comparing the deuterium content in roots obtained from CRM and EA-Cr/HTC-IRMS, it was observed that CRM can be considered a suitable method for such a measurement if the deuterium content is higher than about 1%. In addition to bulk CD%, we reported the spatial distribution pattern of deuterium in root cross-sections by HIM-CRM correlation. Additionally, it was found that by increasing the deuterium content in roots, the lignification decreases.

In the next step, this procedure can be applied in more realistic systems, namely plant roots grown in soil. Without any changes in the workflow, the technique may also be useful to investigate the growth activity in other parts of plants. In order to obtain deeper insight into the deuterium incorporation into the molecular structure of plant tissue the flow of micro-analyses can be extended by time-of-flight secondary ion mass spectrometry. As another field of potential application, it seems possible to employ deuterium labeling in combination with correlative HIM-CRM to study microbial biofilm growth using the here suggested workflow with slight adaptations.

## Methods

### Hydroponic growing of maize plant

Wild-type (WT) maize seeds which were supplied by the Institute of Crop Science and Resource Conservation, University of Bonn, were surface sterilized by ethanol and 10% sodium hypochlorite [56]. Seeds were put on a wet filter paper in darkness at 24 ± 2 °C for 3 days to grow seedlings. Seedlings with a root length of ~ 2–3 cm were put in front of the window and exposed to normal sunlight to reach 1 to 2 leaves (after around 7–10 days) and were watered daily. Then, four plants were transferred to a house-made glass beaker (pots) including 800 ml of half-strength Hoagland solution (HS-HS) and placed in the greenhouse under controlled conditions for 6 days with day and night temperatures of 22 °C and 16 °C, respectively, a light intensity of 80 kLux and relative humidity of 60%. Chemical components and concentrations of the modified Hoagland solution [57] are reported in Additional file 6: Table S3. After that, the solution was replaced with 800ml of new HS-HS in which 40% of the water was deuterated (D_2_O, purity 99.8 atom%, Sigma Aldrich, Germany). Also, in the non-deuterated pot (acting as reference) the solution was replaced with 800ml of normal water HS-HS. The isotope composition of the water in Leipzig is about 0.015 atom%. Two biological replicates were grown in this experiment.

### Root sampling and cross-section preparation

Using a sterile pair of scissors, the primary root was cut into pieces approximately 1 cm in length at three different root zones, namely the young root (Y) as the root tip, the middle (M), and the old part of the root (O). In the whole manuscript the abbreviations D, N, and T refer to deuterated root, non-deuterated root, and the time of harvesting, respectively. The cutting/harvesting was done 1, 2, 3, and 4 days after the replacement of the medium with D_2_O-HS-HS. After cutting, the roots were immediately fixed with 4% paraformaldehyde (PFA) dissolved in 1x phosphate-buffered saline (PBS) for 3 h on ice [58] to ensure slow and gentle penetration of the fixative into the root. Thereafter, they were washed two times with PBS buffer and dehydrated in a graded ethanol series 30%, 50%, 70%, 80%, 90%, and 3 times 100% (v:v), each for 7 min. Finally, to retain the structure of roots during the drying step, the samples were critical point dried (CPD) with a Leica EM CPD 300 (Leica, Austria). Alongside the roots also leaves were cut 1, 2, 3, and 4 days after the incubation with D, left for drying in an oven at 50 °C for 3 days, and then stored in a desiccator until analyzed. A schematic diagram of the preparation steps for roots and leaves is shown in Additional file 5: Fig S3.

For the preparation by vibratome-sectioning for HIM and CRM analysis, each root was placed in a house-made stainless-steel holder and fixed therein using a conductive, vacuum-compatible carbon tape (purchased from Plano GmbH, Wetzlar, Germany). The fixed roots were then cross-sectioned with a Leica VT1200S vibratome (Leica, Austria) using 0.40 mm/s feed speed and 0.60 mm blade amplitude. Prior to HIM analysis the quality of the cross-sections was checked by an optical microscope.

### Elemental analysis-chromium based high temperature conversion-isotope ratio mass spectrometry (EA-Cr/HTC-IRMS)

For hydrogen isotope analysis by EA-Cr/HTC-IRMS, approximately 1 ± 0.1 mg of leaf sample and 1 ± 0.3 mg of root sample, respectively, were weighted into separate tin foil capsules (3.5 mm x 5 mm, HEKAtech, Germany). For the analysis, a HTO high-temperature conversion elemental analyzer (HEKAtech GmbH, Germany) directly coupled via a ConFlo IV open split system (Thermo Fisher Scientific, Germany) to a MAT 253 isotope ratio mass spectrometer (Thermo Fisher Scientific, Germany) was used. The reactor temperature was kept at 1200 °C and helium (He 5.0) was used as carrier gas with a flow rate of 75 mL/min. The samples were applied by an auto-sampler (AS200, CE Instruments, Italy). The deuterium isotope composition is reported in atom %; the concentration of deuterium in the non-labeled biomass was determined to be about 0.02 atom%. A detailed description of the experimental setup can be found elsewhere [59, 60]. Samples were analyzed in replicates and the analytical precision (STDEV) for leaves and roots was below ± 3.097 and ± 1.604 atom%, respectively. The high standard deviation can be attributed to the non-homogeneity of the samples.

### Helium ion microscopy (HIM)

For structural analysis of the vibratome-cut root sections a Zeiss Orion NanoFab scanning helium-ion microscope (Carl Zeiss Microscopy, Peabody, MA, USA) was used. The ion landing energy and beam current amounted to 25 kV and approximately 0.5pA, respectively. For imaging secondary electrons were detected by an Everhardt-Thornley-type electron detector. Charge compensation was carried out with an electron flood-gun which was turned on after the scan of each line and irradiated the field-of-view with 670 eV electrons for 300µs. To avoid flood-gun electrons to reach the electron detector the scan was delayed for 300µs before the next line was scanned. Depending on the diameter of the root sections quadratic images with edge length of 500 to 800 μm were scanned at a 2048$\times$2048 pixel^2^ raster. The beam dwell time ranged from 0.5µs to 2µs, depending on the obtained contrast for the particular sample. For noise reduction each line was scanned 16 times in line-averaging mode.

### Confocal Raman micro-spectroscopy (CRM)

CRM on the root cross-sections was performed with a WITec alpha300 confocal Raman microscope (WITec GmbH, Ulm, Germany). The field-of-view for the two-dimensional Raman mapping was chosen with regard to the structural integrity of the cross-sections based on the previously acquired HIM images. All CRM measurements were carried out with a 20x Zeiss EC Epiplan-Neofluar air objective with a numerical aperture (NA) of 0.5. The excitation light source was a 532 nm solid-state laser (assembled by WITec GmbH, Ulm, Germany), and the power on the sample was set to 5 ± 0.5 mW. The Raman-scattered light was passed through an optical fiber (diameter of 50 μm) into a grating monochromator with a 600 g/mm grating (UHTS 300, WITec GmbH, Ulm, Germany), and then detected by a charged coupled device camera (DU401A-BR-DD-352, Andor, EU). The set-up is controlled by the software Control FIVE, Tec Suite 5.2 (WITec GmbH, Ulm, Germany). During the CRM analysis strips of 400 to 600 μm length and 50 μm width were scanned at a pixel size of 2 μm using an integration time of 10 s. The theoretical lateral resolution of the system in the described configuration is 650 nm (based on $0.61\lambda$/NA [61]). In order to compensate for the topography of the root-section surfaces, the optical-profilometer-like add-on WITec TrueSurface was enabled to keep the surface within the focus plane during the entire analysis. The obtained CRM data were then processed using the WITec Project FIVE software (5.3.10.102 plus version).

To verify whether the calculation of CRM data is precise, different concentrations of deuterated glucose (D-glucose, 97 atom% D, Sigma Aldrich, Germany) was prepared with 0 (natural abundance) to 40% (v:v) concentrations. Then, 5 µl of each solution was added on a calcium fluoride (CaF_2_) window, Raman grade, 10 mm*0.5 mm, optically polished (Korth Kristalle, Germany) and let it to be dried and measured by Raman with the same parameters as described above.

### Correlative analysis and image registration

In order to combine the high-resolution structural information obtained from HIM analysis with the chemical maps obtained from CRM - in particular the distribution of the deuterium label in the sample - these correlatively acquired image data were registered onto each other. Using the HIM data as *target* and the CRM maps as *moving images* the image registration was carried out with the FIJI-based plug-in *Correlia* [40]. Due to the low structural similarity of the HIM and the CRM images intensity-based automatic registration was not possible. Instead, manually selected landmarks were matched.

## Electronic supplementary material

Below is the link to the electronic supplementary material.

**Additional file 1: Fig. S1**. Deuterium content in sample (atom%) vs. CRM measured (CD%) of deuterated glucose samples. L-range (light blue) and A-range (dark blue) refer to the integration ranges for the CD and CH bands according to literature (L) (2040-2300 cm^-1^ for CD and 2800-3100 cm^-1^ for CH) and acquired data in this work (A) (2033-2303 cm^-1^ for CD and 2665-3045 cm^-1^ for CH). Both L and A-ranges demonstrated the linearity of the method.

**Additional file 2: Fig. S2**. CD (%) in roots measured by CRM based on L and A-range. The literature (L) range was between 2040 and 2300 cm^− 1^ for CD and 2800-3100 cm^− 1^ for CH and the acquired data in this work (A) range was between 2093 and 2309 cm^− 1^ for CD and 2779-3075 cm^− 1^ for CH bands. Blue circles show close CD% calculated by L and A-ranges and yellow circles represent different CD% calculated using these two ranges.

**Additional file 3: Table S1**. CD% values of root samples measured by CRM and error in the calculations (ΔCD%). The determination of the area of the CD band in the Raman spectra was done by two different integration ranges. Literature (L-range) was from 2040 to 2300 cm^− 1^ for the CD band and 2800-3100 cm^− 1^ for the CH band. Acquired data in this work (A-range) was from 2093 to 2309 cm^− 1^ for the CD band and 2779-3075 cm^− 1^ for the CH band. A negative value (e.g., YDT3-2) shows bad-quality data with strong auto-fluorescence. The error in the calculations (ΔCD%) was also calculated using both ranges.

**Additional file 4: Table S2**. Laboratory-prepared vs. CRM measured deuterium content (%) in deuterated glucose samples. The determination of deuterium content was done by the integration range applied in literature (L-range) from 2040 to 2300 cm^− 1^ for the CD band and 2800-3100 cm^− 1^ for the CH band and acquired data in this work (A-range) from 2033 to 2303 cm^− 1^ for the CD band and 2665-3045 cm^− 1^ for the CH band. The error in the calculations (ΔCD%) was also calculated using both ranges.

**Additional file 5: Fig. S3**. Schematic diagram of the preparation of maize roots & leaves. Roots were grown hydroponically in Hoagland solution containing 40% D_2_O. Primary roots were harvested in young (Y), middle (M), and old (O) root zones and subsequently chemically fixed. Roots were then analyzed by EA-Cr/HTC-IRMS or were cut by vibratome, imaged by HIM and analyzed by CRM. Leaves were harvested in the tip (T), center (C), and base (B) zones, oven-dried at 50 °C and analyzed by EA-Cr/HTC-IRMS.

**Additional file 6: Table S3**. Chemical composition of Hoagland solution used in this study.

## Data Availability

The datasets generated and/or analyzed during the current study are available in the UFZ Data Investigation Portal (https://www.ufz.de/record/dmp/archive/13952) repository.
