# Supplementary figures and images for: Tracking deuterium uptake in hydroponically grown maize roots using correlative helium ion microscopy and Raman micro-spectroscopy
Source: Plant Methods. 2023 Jul 14;19:71. doi: 10.1186/s13007-023-01040-y (PMC10347822; doi:10.1186/s13007-023-01040-y)

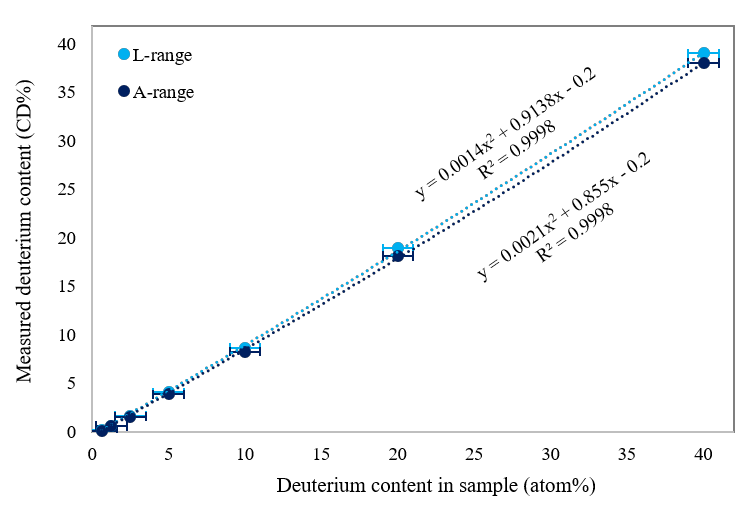

Supplement: Supplementary file 1 — Additional file 1: Fig. S1. Deuterium content in sample (atom%) vs. CRM measured (CD%) of deuterated glucose samples. L-range (light blue) and A-range (dark blue) refer to the integration ranges for the CD and CH bands according to literature (L) (2040-2300 cm-1 for CD and 2800-3100 cm-1 for CH) and acquired data in this work (A) (2033-2303 cm-1 for CD and 2665-3045 cm-1 for CH). Both L and A-ranges demonstrated the linearity of the method. [file 13007_2023_1040_MOESM1_ESM.png]

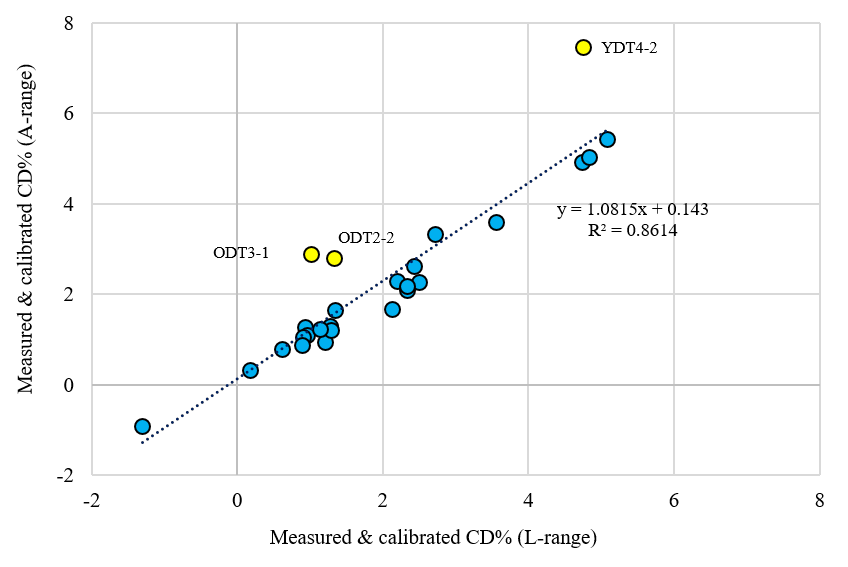

Supplement: Supplementary file 2 — Additional file 2: Fig. S2. CD (%) in roots measured by CRM based on L and A-range. The literature (L) range was between 2040 and 2300 cm− 1 for CD and 2800-3100 cm− 1 for CH and the acquired data in this work (A) range was between 2093 and 2309 cm− 1 for CD and 2779-3075 cm− 1 for CH bands. Blue circles show close CD% calculated by L and A-ranges and yellow circles represent different CD% calculated using these two ranges. [file 13007_2023_1040_MOESM2_ESM.png]

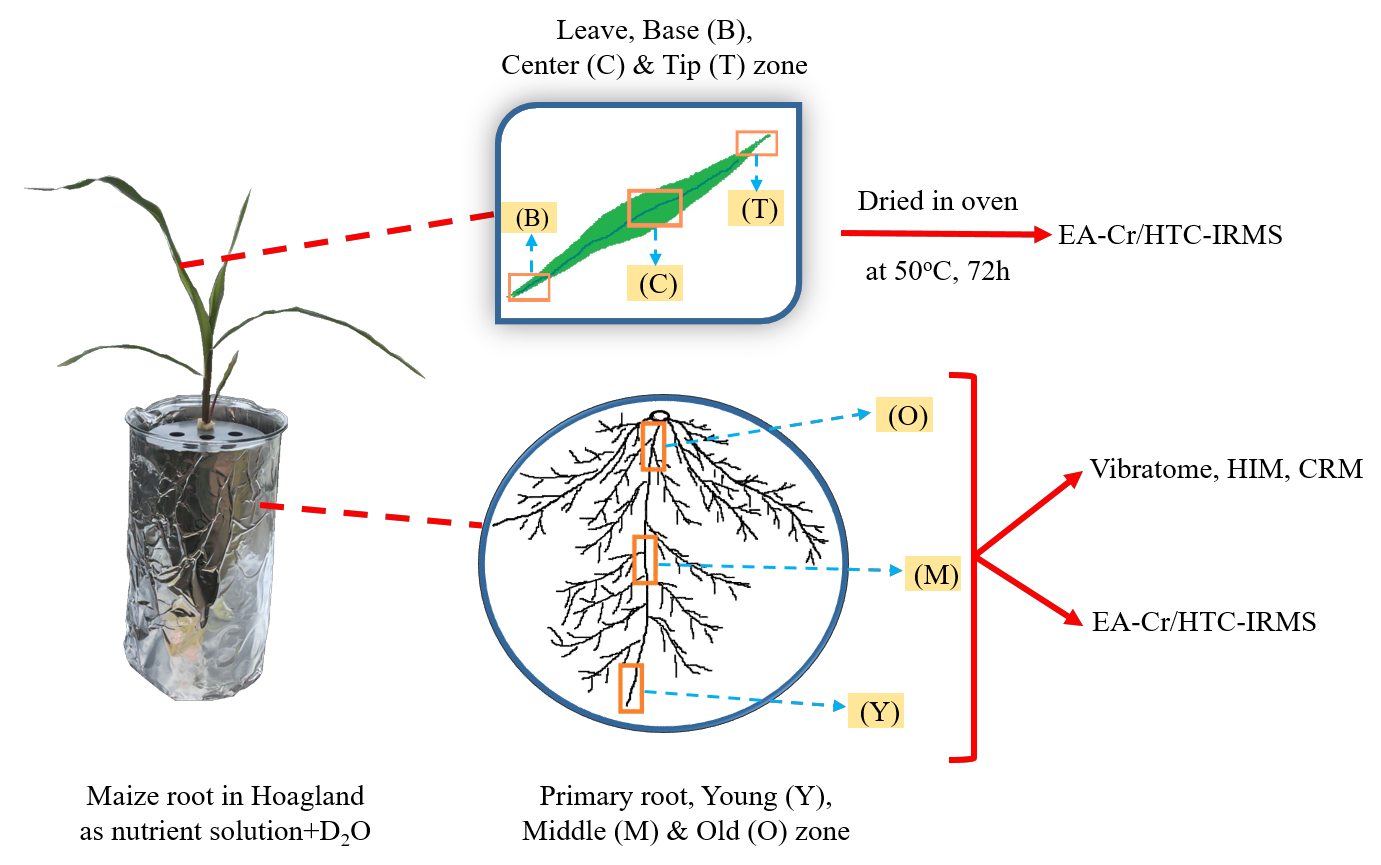

Supplement: Supplementary file 5 — Additional file 5: Fig. S3. Schematic diagram of the preparation of maize roots & leaves. Roots were grown hydroponically in Hoagland solution containing 40% D2O. Primary roots were harvested in young (Y), middle (M), and old (O) root zones and subsequently chemically fixed. Roots were then analyzed by EA-Cr/HTC-IRMS or were cut by vibratome, imaged by HIM and analyzed by CRM. Leaves were harvested in the tip (T), center (C), and base (B) zones, oven-dried at 50 °C and analyzed by EA-Cr/HTC-IRMS. [file 13007_2023_1040_MOESM5_ESM.png]
