# Supplementary material for: Tracking deuterium uptake in hydroponically grown maize roots using correlative helium ion microscopy and Raman micro-spectroscopy
Source: Plant Methods. 2023 Jul 14;19:71. doi: 10.1186/s13007-023-01040-y (PMC10347822; doi:10.1186/s13007-023-01040-y)
Supplement: Supplementary file 3 — Additional file 3: Table S1. CD% values of root samples measured by CRM and error in the calculations (ΔCD%). The determination of the area of the CD band in the Raman spectra was done by two different integration ranges. Literature (L-range) was from 2040 to 2300 cm− 1 for the CD band and 2800-3100 cm− 1 for the CH band. Acquired data in this work (A-range) was from 2093 to 2309 cm− 1 for the CD band and 2779-3075 cm− 1 for the CH band. A negative value (e.g., YDT3-2) shows bad-quality data with strong auto-fluorescence. The error in the calculations (ΔCD%) was also calculated using both ranges. [file 13007_2023_1040_MOESM3_ESM.pdf]

Table S1 CD% values of root samples measured by CRM and error in the calculations (ΔCD%)

Root samples	CD band integration limit (L-range)	CD band integration limit (A-range)
	**CD%±ΔCD% (error)**	**CD%±ΔCD% (error)**
YN	0.94±5.67	1.28±5.18
MN	1.27±1.39	1.29±1.35
ON	1.20±2.42	0.95±2.29
YDT1-1	2.20±1.44	2.30±1.40
YDT1-2	2.72±1.57	3.33±1.51
MDT1-1	1.28±1.66	1.31±1.59
MDT1-2	1.29±1.82	1.21±1.75
ODT1-1	2.33±2.62	2.10±2.51
ODT1-2	0.62±1.31	0.79±1.28
YDT2-1	4.73±2.31	4.93±2.19
YDT2-2	3.56±1.33	3.60±1.31
MDT2-1	2.44±3.23	2.63±3.02
MDT2-2	0.96±1.42	1.10±1.38
ODT2-1	2.13±2.79	1.68±2.66
ODT2-2	1.33±3.69	2.79±3.34
YDT3-1	0.91±1.65	1.06±1.59
YDT3-2	-1.31±2.21	-0.92±2.08
MDT3-1	1.34±1.82	1.66±1.75
MDT3-2	0.89±1.04	0.89±1.03
ODT3-1	1.01±3.38	2.89±3.03
ODT3-2	1.14±1.45	1.23±1.41
YDT4-1	5.07±1.39	5.44±1.35
YDT4-2	4.76±4.20	7.47±3.62
MDT4-1	2.50±2.62	2.27±2.50
MDT4-2	2.34±2.51	2.18±2.39
ODT4-1	0.17±1.13	0.34±1.11
ODT4-2	4.83±5.44	5.03±5.07

The determination of the area of the CD band in the Raman spectra was done by two different integration ranges. Literature (L-range) was from 2040-2300cm^{-1} for the CD band and 2800-3100cm^{-1} for the CH band. Acquired data in this work (A-range) was from 2093-2309cm^{-1} for the CD band and 2779-3075cm^{-1} for the CH band. A negative value (e.g., YDT3-2) shows bad-quality data with strong auto-fluorescence. The error in the calculations (ΔCD%) was also calculated using both ranges.
