# Supplementary material for: Tracking deuterium uptake in hydroponically grown maize roots using correlative helium ion microscopy and Raman micro-spectroscopy
Source: Plant Methods. 2023 Jul 14;19:71. doi: 10.1186/s13007-023-01040-y (PMC10347822; doi:10.1186/s13007-023-01040-y)
Supplement: Supplementary file 4 — Additional file 4: Table S2. Laboratory-prepared vs. CRM measured deuterium content (%) in deuterated glucose samples. The determination of deuterium content was done by the integration range applied in literature (L-range) from 2040 to 2300 cm− 1 for the CD band and 2800-3100 cm− 1 for the CH band and acquired data in this work (A-range) from 2033 to 2303 cm− 1 for the CD band and 2665-3045 cm− 1 for the CH band. The error in the calculations (ΔCD%) was also calculated using both ranges. [file 13007_2023_1040_MOESM4_ESM.pdf]

Table S2 Laboratory-prepared vs CRM measured deuterium content (%) in deuterated glucose samples

**The concentration of laboratory-prepared D-glucose (atom%)**	**CD%±ΔCD% (error) measured by CRM & calculated by CD band integration limit**	
	**L-range**	**A-range**
0	0.06±0.11	0.06±0.10
0.62	0.36±0.06	0.34±0.06
1.25	0.84±0.14	0.80±0.13
2.5	1.88±0.08	1.78±0.08
5	4.28±0.09	4.06±0.09
10	8.88±0.10	8.48±0.10
20	19.11±0.08	18.34±0.08
40	39.20±0.02	38.22±0.02

The determination of deuterium content was done by the integration range applied in literature (L-range) from 2040-2300cm^{-1} for the CD band and 2800-3100cm^{-1} for the CH band and acquired data in this work (A-range) from 2033-2303cm^{-1} for the CD band and 2665-3045cm^{-1} for the CH band. The error in the calculations (ΔCD%) was also calculated using both ranges.
