# Supplementary material for: Tracking deuterium uptake in hydroponically grown maize roots using correlative helium ion microscopy and Raman micro-spectroscopy
Source: Plant Methods. 2023 Jul 14;19:71. doi: 10.1186/s13007-023-01040-y (PMC10347822; doi:10.1186/s13007-023-01040-y)
Supplement: Supplementary file 6 — Additional file 6: Table S3. Chemical composition of Hoagland solution used in this study. [file 13007_2023_1040_MOESM6_ESM.pdf]

Table S3 Chemical composition of Hoagland solution used in this study

Type of nutrients	Chemical	Mass to prepare stock solution (g/100ml)	Concentration (ml/L)
Macronutrients	$Ca(NO_3)_2.4H_2O$	23.62	5
	KNO_3	10.11	5
	$MgSO_4.7H_2O$	24.65	2
	KH_2PO_4	13.61	1
Micronutrients	H_3BO_3	0.28	1
	$MnCl_2.4H_2O$	0.18	
	$CuSO_4.5H_2O$	0.008	
	$ZnSO_4.7H_2O$	0.023	
	$Na_2MoO_4.2H_2O$	0.002	
Fe-EDTA	$FeSO_4.7H_2O$	0.56	2.5
	Na_2EDTA	0.74	
